# Ginsenosides: Allies of gastrointestinal tumor immunotherapy

**DOI:** 10.3389/fphar.2022.922029

**Published:** 2022-10-31

**Authors:** Yutao Feng, Fen Ma, Enjiang Wu, Zewei Cheng, Zhengtao Wang, Li Yang, Jiwei Zhang

**Affiliations:** Shanghai Key Laboratory of Compound Chinese Medicines, The MOE Key Laboratory for Standardization of Chinese Medicines, Institute of Chinese Materia Medica, Shanghai University of Traditional Chinese Medicine, Shanghai, China

**Keywords:** traditional Chinese medicine, ginseng, ginsenosides, gastrointestinal tumors, immunotherapy

## Abstract

In the past decade, immunotherapy has been the most promising treatment for gastrointestinal tumors. But the low response rate and drug resistance remain major concerns. It is therefore imperative to develop adjuvant therapies to increase the effectiveness of immunotherapy and prevent drug resistance. Ginseng has been used in Traditional Chinese medicine as a natural immune booster for thousands of years. The active components of ginseng, ginsenosides, have played an essential role in tumor treatment for decades and are candidates for anti-tumor adjuvant therapy. They are hypothesized to cooperate with immunotherapy drugs to improve the curative effect and reduce tumor resistance and adverse reactions. This review summarizes the research into the use of ginsenosides in immunotherapy of gastrointestinal tumors and discusses potential future applications.

## Introduction

Gastrointestinal (GI) cancers are one of the most common malignant tumors globally, with a high incidence and mortality rate (Sung et al., 2021). Despite significant advances in disease diagnosis and drug development, GI cancers remain the leading cause of cancer death; thus, it is imperative to develop novel therapeutic approaches for patients affected by these cancers. The current treatment methods for GI cancers generally include adjuvant chemotherapy, chemoradiation, surgical resection, and perioperative chemotherapy. Among these, chemotherapy and radiotherapy are the first-line treatments for advanced GI tumors, and they can only prolong patients’ survival. Tumors easily develop strong resistance to chemotherapy drugs, with a high recurrence and metastasis rate, and patients have a poor overall prognosis (Baxter et al., 2021). Currently, immunotherapy is one of the most promising therapies to overcome these difficulties (Gourd, 2021).

Immunotherapy, which has recently emerged as a new beneficial therapy, relies on the immune system to attack and eliminate tumors. The first attempts to stimulate active host immunity were based on administration of cytokines, particularly interferon-γ, interleukin-2 (IL-2), IL-10, and GM-CSF. The use of immune checkpoint inhibitors has gradually gained the attention of the medical community (Kirkwood et al., 2012). The anti-CTLA-4 antibody ipilimumab and the anti-PD-1mAbs pembrolizumab and nivolumab were first approved by the United States FDA to treat metastatic melanoma in 2011 and 2014, respectively. Four years later, Dr. James P. Allison and Dr. Tasuku Honjo were awarded the 2018 Nobel Prize in Physiology or Medicine for their work on immune checkpoint therapy in cancer (Altmann, 2018). It is expected that if these antibodies are used to target the immune escape mechanisms, various cancers will be controlled or even cured. The FDA and EMA have also approved the use of immunotherapy for melanoma, lung cancer, head and neck squamous cell carcinoma, renal cell carcinoma, and other tumors (Gong et al., 2018). However, there are still many challenges to overcome. Immune checkpoint inhibitors are effective for treating tumors with high mismatch repair ability or microsatellite instability but are ineffective on microsatellite stable tumors. About 95% of colorectal cancers are microsatellite stable, and these patients cannot benefit from immunotherapy. Other challenges are low tumor mutation pressure and lack of immune cell infiltration, which are hypothesized to be the main mechanisms of immune resistance (Chan et al., 2019; Samstein et al., 2019). A variety of small molecules (Xu et al., 2021; Jiang et al., 2022) and targeted inhibitors (Li et al., 2019a; Lee and Konstantinopoulos, 2019; Wang et al., 2022; Zhang et al., 2022) can improve immunotherapy, and screening for boosters for immunotherapy has become a research hotspot.

Ginseng, which is the dried root or rhizome of Panax ginseng C.A. Mey., a plant of the Araliaceae family, is a potential source of such boosters. It is listed as “top grade” (i.e., a non-toxic medicinal grade) in *Shen Nong’s Materia Medica* and has been used in traditional medical clinics for thousands of years. It is believed to nourish the original Qi, replenish vital energy and body fluid, promote growth, soothe the nerves, and nourish the mind (Gao, 1990). Ginseng preparations have been widely used in medicine and health care in Asia and Europe (Rubio et al., 2018; Chen et al., 2020). Ginseng is rich in bioactive ingredients such as triterpene saponins and polysaccharides. Among these bioactive ingredients, the main components are ginsenosides, which have a wide range of pharmacological effects, such as anti-tumor (Linming et al., 2017), anti-oxidation (He et al., 2022), and anti-aging (Wang et al., 2021a) activities. They also protect the liver (Qu et al., 2021; Li et al., 2022a) and the cardiovascular and cerebrovascular systems (Zhang et al., 2019; Han et al., 2022a; Ni et al., 2022). This review describes the research progress in the use of ginsenosides in various tumor treatments with an emphasis on their use in immunotherapy of GI tumors.

## Ginseng components are a candidate source of anti-tumor adjuvants

Ginseng is the most commonly used herbal medicine worldwide. Many scholars believe that ginseng can be used to identify drug candidates for adjuvant tumor treatment (Yun, 2001; Wong et al., 2015; Majeed et al., 2018). Recent studies have explored the anti-tumor activities of various components of ginseng and their synergistic anti-cancer effects.

### Effects of ginseng components on immunity

**FIGURE 1:**
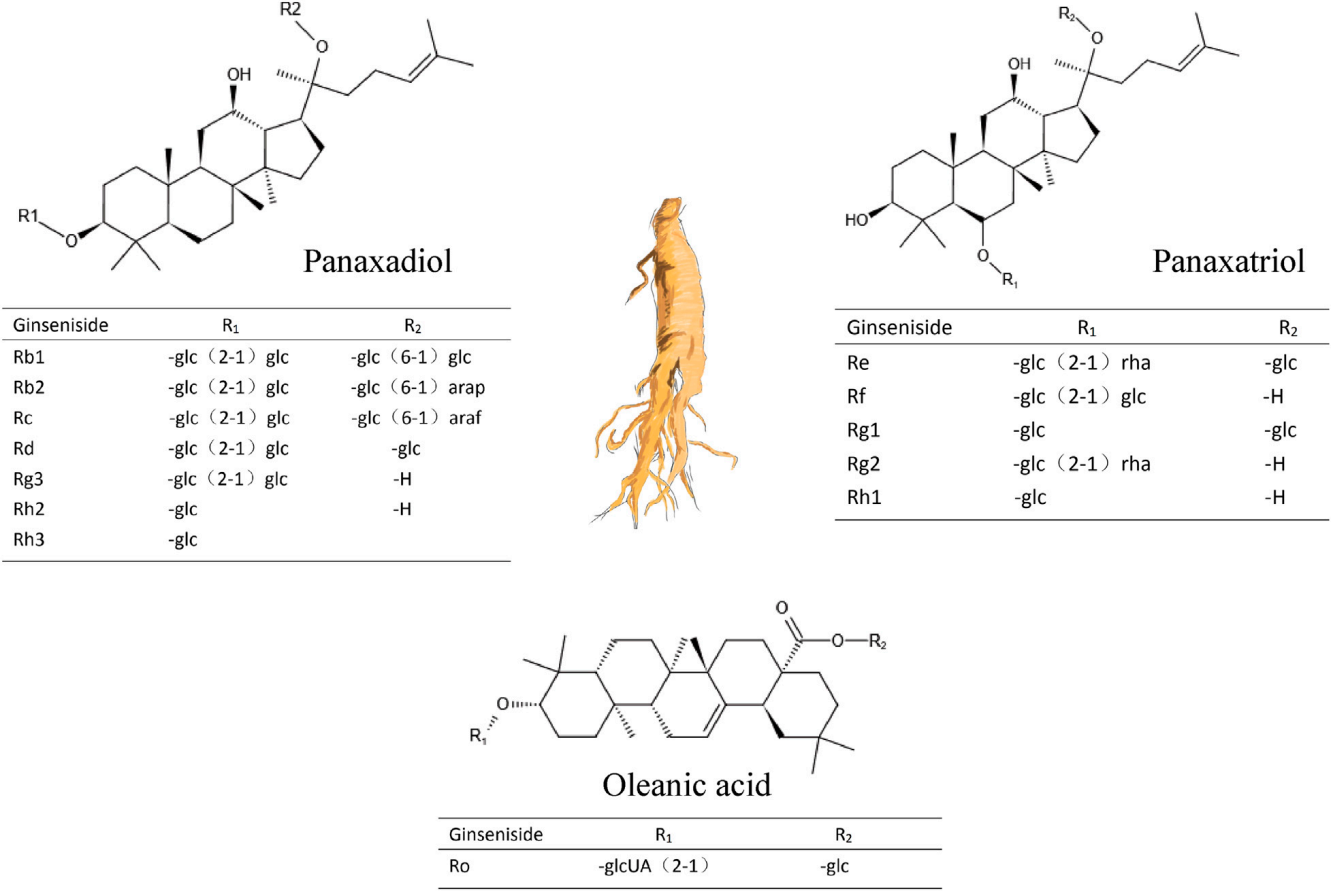
Three types of ginsenosides and their chemical structures.

Ginsenosides are the main components of ginseng, and they are categorized as protopanaxadiol-, protopanaxatriol, and oleanolic acid-type ginsenosides (Figure 1 and Table 1) based on the structure of the aglycon (Hou et al., 2021). To date, more than 100 types of ginsenosides have been isolated (Jia and Zhao, 2009; Wang et al., 2021b).

**TABLE 1:** The effects of ginseng components on immunity.

Ginseng component	Model	Pharmacology	Therapeutic dose	Pharmacological mechanism	References
KG-135	HeLa cervical cancer cell line	Enhancement of HeLa cell apoptosis induced by etoposide	0–75 μg/ml	Bax and p21^Waf1/Cip1^↑; G1 cyclin-dependent kinase↓; p53^Ser−15^↓; p-p53^Ser−15^↓	Lee et al. (2010)
Rg3	CTX-treated mice	Reversal of CTX-induced immunosuppression	7.5/15 mg/kg	CD3^+^ ↑ CD4^+^↑	Liu et al. (2019)
F2	Alcoholic liver injury mouse model	Attenuates chronic-binge ethanol-induced liver injury	50 mg/kg	IL-10↑; T_reg_ cells↑; IL-17↓; Th17 cells↓	Kim et al. (2020a)
20S-protopanaxadiol	ER^+^ MCF-7 and ER^−^ MDA-MB231 cell line	Estrogen-stimulated MCF-7 cell inhibition; enhancement of tamoxifen cytotoxicity	5 μM/10 μM	estrogen-stimulated gene expression ↓	Yu et al. (2007)
Rg5	Nude mice bearing A549/T tumors	Preventing tumor drug resistance	1–100 μM	p-Akt↓, Nrf2↓,	Feng et al. (2020)
GPs	K562, HL-60, KG1α cells	Mouse peritoneal macrophage-mediated cytotoxicity	0, 25, 50, 100, and 200 mg/L	TNF-α↑, IL-1↑, IL-6↑, NO↑	Wang et al. (2010)
Re3/Rk	Balb/c mice myelo-suppression model	Peripheral blood cells↑, bone marrow nucleated cell counts↑, thymus and spleen index↑	5/10 mg/kg	Bcl-2↑; bax↓caspase-3↓	Han et al. (2019)

The immunomodulatory effects of ginseng components have been widely reported, and we review the pharmacological effects they have on the immune system. Ginseng KG-135 preparation, a mixture of three ginsenosides, Rk1, Rg,3, and Rg5, inhibits the phosphorylation of proteins in the p53 pathway, and thus enhances etoposide-induced HeLa cell apoptosis (Lee et al., 2010). Ginsenoside F2 regulates immunity by increasing IL-10 expression and the number of regulatory T cells while decreasing IL-17 expression and the number of Th17 cells (Kim et al., 2020a). Ginsenoside Rg5 targets the ABCB1 transporter and is combined with docetaxel to prevent drug resistance (Feng et al., 2020). Additionally, another active ingredient in ginseng, 20 S-protopanaxadiol, was found to inhibit the growth of ER-positive breast cancer cells and enhance tamoxifen cytotoxicity (Yu et al., 2007). Rg3 was shown to have a protective effect on immunosuppression induced by cyclophosphamide (CTX) (Liu et al., 2019), which is used as a broad-spectrum anti-tumor chemotherapy drug combined with immunotherapy to treat metastatic breast cancer in humanized mouse bone marrow (Roghanian et al., 2019). Rg3 was found to further reduce immune capacity after CTX injury. Another study showed that the ginsenosides Re and Rk3 can relieve symptoms of bone marrow suppression and improve bone marrow hematopoietic function, regulate bcl-2/bax balance, and inhibit the caspase-3 expression, preventing apoptosis of bone marrow nucleated cells (Han et al., 2019). When ginseng polysaccharides were used to treat mouse peritoneal macrophages, they were clearly cytotoxic to K562, HL-60, and KG1 cells (Wang et al., 2010).

Various components in ginseng often play different roles in immune regulation. In Traditional Chinese medicine (TCM) different combinations of herbs are chosen to prepare water decoctions that are prescribed to treat patients (Gao, 1990). In TCM, ginsenosides play a major role in T cell development, cytokine secretion, helper T cell function, and other putative signal pathways to enhance the total immune system. But in modern pharmacological studies, results of studies in which ginseng components are administered alone have disappointing. Even when used as immune adjuvants, they tend to work at higher concentrations than common small molecule drugs, which has led researchers to worry about their toxicity to the liver and kidney (Wang et al., 2010; Kim et al., 2020a). Based on the current reports, we believe that only a few components of ginseng have strong pharmacological activity and have the potential to be developed as small molecule drugs for chemotherapy. Instead, the vast majority of ginseng components have immune regulation functions and may be effective as immune adjuvants. Furthermore, ginseng components can affect to several known drug resistance mechanisms (De Vera et al., 2019; Bu et al., 2022; Shurin and Umansky, 2022). Their probable benefits to immunotherapy deserve our attention.

### The effects of ginseng components on immune checkpoint blockade

Increasing evidence suggests that ginseng components may improve immunotherapy. In mice, ginseng polysaccharides were found to increase the anti-tumor responses to αPD-1 mAb by increasing the content of the microbial metabolite valeric acid and the Kynurenine/Tryptophan ratio. The therapeutic effect of ginseng polysaccharide contributes to the suppression of regulatory T cells and induction of effector T cells after this combination treatment (Han et al., 2022b). In an A549 xenograft mice model, ginsenosides Rk1 and Rg3 significantly inhibited tumor growth and reduced PD-L1 expression by blocking NF-κB signaling (Jiang et al., 2017; Hu et al., 2020), and they induced MDA-MB-231 cell apoptosis by blocking PI3K/Akt signaling (Hong and Fan, 2019). Because ginsenosides have high lipophilicity, researchers believe that they may be suitable for use as targeting vectors in addition to being used as drugs (Han et al., 2022b). The lipophilic structure of ginsenoside Rg3 is advantageous for packaging into liposomes for docetaxel delivery. Docetaxel delivered *via* Rg3 promoted tumor immunity by reversing the formation of an immunosuppressive microenvironment in triple-negative breast cancer (Xia et al., 2022). Ginseng-derived nanoparticles can reprogram tumor-associated macrophages in colon cancer to increase CCL5 and CXCL9 secretion and recruitment of CD8^+^ T cells into the tumor bed; these nanoparticles synergized with PD-1 mAb therapy with no detected systemic toxicity (Han et al., 2022b). STAT3 and PD-L1 are critically involved in cancer proliferation (Bu et al., 2017; Jiang et al., 2019; Zou et al., 2020), progression, and immunosuppression. The ginseng saponin metabolite, compound K, inhibits PD-L1 and STAT3 signaling in prostate cancer cells by activating miR193a-5p (Lee et al., 2021). The above attempts to use ginsenosides to increase the effectiveness of immunotherapy have achieved some success.

**FIGURE 2:**
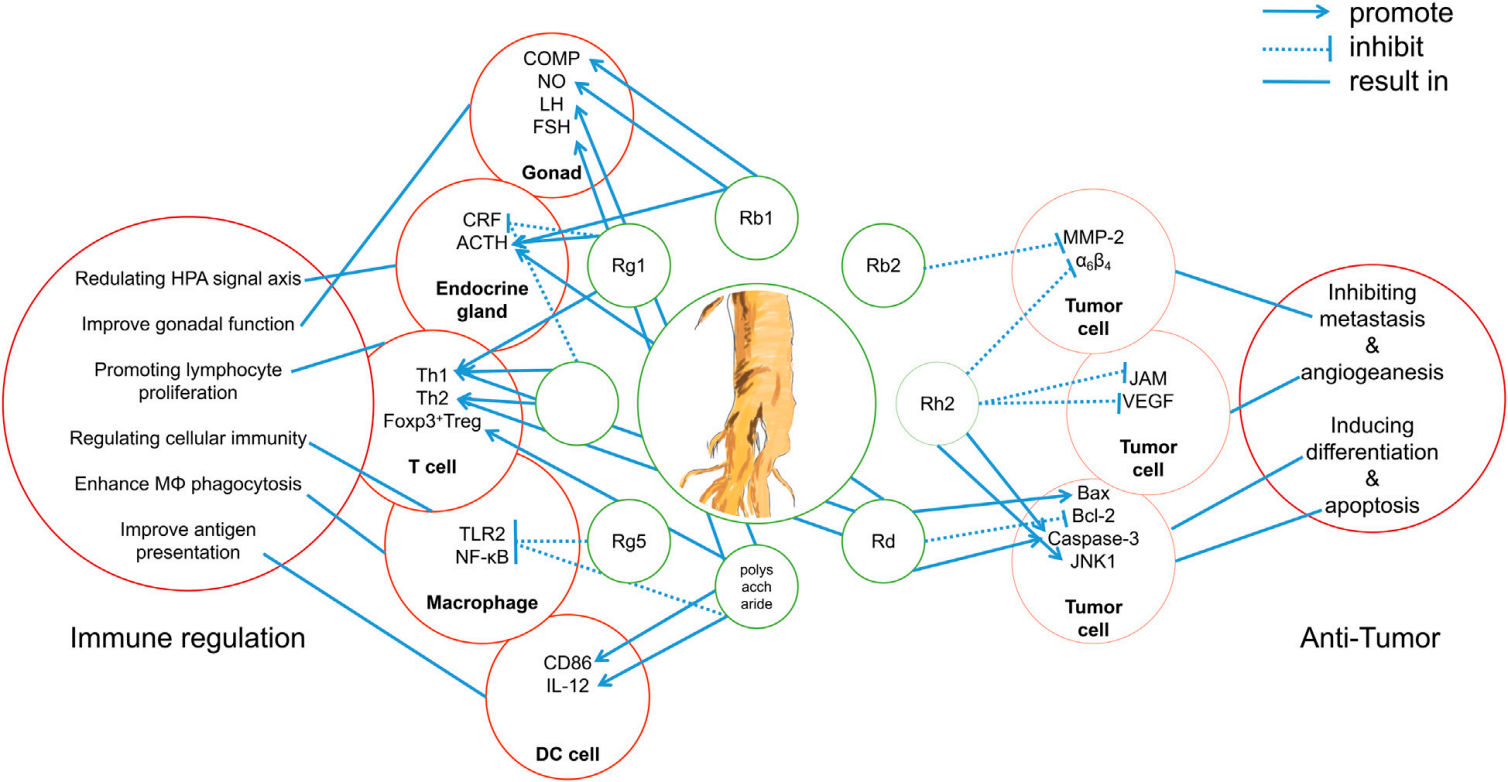
Integrated mechanisms of active components of ginseng.

In summary, several studies have shown that ginseng components have great potential in immunotherapy, and the main components of ginseng, ginsenosides, remain a hot topic of research, with studies exploring their anti-tumor effects and mechanisms. However, through reviewing previous studies, we also found many common problems in these studies. In the past 20 years, traditional molecular biology methods have been used to study ginseng compounds, and researchers have stopped at verifying the signal pathways affected by ginsenosides and the pharmacological effects *in vivo* (Figure 2). However, how ginsenosides affect the tumor immune microenvironment remains unknown. This dilemma is due to the lack of identification of pharmacological targets and biomarkers in the study of ginsenosides. Technologies such as co-immunoprecipitation, surface plasmon resonance, and microscale thermophoresis are in the forefront of those being used to identify TCM targets (Xu et al., 2021b; Gu et al., 2022). The use of molecular interaction technology to identify the pharmacological targets of ginsenosides will help to deepen our understanding of the pharmacology of ginsenosides.

## Research progress in the use of ginsenosides in immunotherapy of digestive tract tumors

### Research progress in the use of ginsenosides in liver cancer immunotherapy

Liver cancer is one of the six most common cancers in the world, and it is often the final outcome of all liver diseases (Gravitz and Pincock, 2014; Sung et al., 2021). The World Health Organization estimates that in 2030, more than 1 million patients will die of liver cancer per year. In the United States, the age-adjusted death rate from liver cancer increased by 43% between 2000 and 2016, from 10.5 per 100,000 U.S. standard population to 15.0 for men. The 5-year survival rate of liver cancer is 20%, making it the second most fatal tumor after pancreatic cancer (Siegel et al., 2022). Furthermore, the prevalence of liver cancer is still rising, and treatment methods are still lacking. Currently liver cancer is mainly treated using physical methods, such as chemotherapy, liver transplantation, and surgery, but researchers are actively exploring new drug targets and therapies (such as immunotherapy) (Centers for Disease Control and Pre-vention, 2018).

The earliest research on ginsenosides as tumor inhibitors for liver cancer can be traced back to a report published in 1997. Park et al. (1997) reported that ginsenoside Rh2 promotes apoptosis of human liver hepatoma SK-HEP-1 cells and subsequent PARP cleavage. PARP is responsible for mediating post-translational PARylation of substrate proteins involved in transcription and DNA damage repair (Noordermeer and van Attikum, 2019). PARP inhibitor (PARPi) inhibits the enzymatic activity of both PARP1 and PARP2, prevents PARylation, and causes the accumulation of DNA single-strand breaks. Therefore, PARPi is used to induce cell death in tumors with homologous recombination (HR) deficiency (Murai et al., 2012; Murai et al., 2014). Ginsenoside Rg1 was reported to inhibit the expression of a key DNA double-strand break repair factor and CtBP interacting protein, thereby impairing HR (Zhen et al., 2018). Chemical sensitivity analysis showed that the combination of Rg1 and olaparib, a first-line PARPi, improves the therapeutic effect of hepatoblastoma treatment. This indicates that Rg1 significantly enhances the sensitivity to DNA damage therapy and that it may be a sensitizer of PARPi.

NHE1 protein is an important therapeutic target for hepatocellular carcinoma (HCC) (Yang et al., 2010). Ginsenoside Rg3 was reported to downregulate NHE1 *in vivo* and *in vitro*, revealing it as an effective multi-target anti-tumor drug for the treatment of HCC. Epidermal growth factor (EGF) can significantly up-regulate the expression of NHE1 and increase the level of phosphorylated extracellular signal-regulated protein kinase (ERK1/2) and the expression of hypoxia-inducible factor 1α (HIF-1α). Rg3 was found to significantly reduce the expression of EGF, EGF receptor (EGFR), phosphorylated ERK1/2, and HIF-1α, indicating that it reduces the expression of NHE1 through overall inhibition of the EGF-EGFR-ERK1/2-HIF-α signaling pathway in HCC (Li et al., 2018).

A Korean study showed that ginsenosides Rb1, Rg3, and Rk1 reduce lipid accumulation and enhance the antioxidant function of the liver both *in vitro* and *in vivo* through the ER stress pathway (An et al., 2021). The source of these compounds, Korean Red Ginseng, could enhance the immune activity of NK cells *via* IL-33 by increasing the number of eosinophils (Kwon et al., 2021).

### Research progress in the use of ginsenosides in immunotherapy of colorectal cancer

Colorectal cancer (CRC) is the leading cause of death after lung cancer, and one of the primary sources of cancer deaths worldwide (Siegel et al., 2022). Early screening has been shown to improve the 5-year survival rate of CRC patients, and the morbidity and mortality rates have shown a downward trend in recent years. Nevertheless, the prognosis of patients with metastatic CRC is still poor. In the United States, the median 5-year survival rate of these patients is only 12.5% (Siegel et al., 2014; Montminy et al., 2019). Therefore, there is a need to develop more treatments for CRC.

In the past 10 years, immunotherapy has successfully achieved long-term tumor suppression for refractory tumors (such as melanoma and non-small cell lung cancer). Hence, there are high hopes for the performance of these therapies in CRC.

Ginsenosides affect many factors responsible for the effects of immunotherapy. Ginsenoside Rh2 enhances the cytotoxicity of 5-fluorouracil (5-FU) in drug-resistant CRC cells (LoVo/5-FU and HCT-8/5-FU) and attenuates the expression of drug-resistance genes, such as MRP1, MDR1, LRP, and GST (Liu et al., 2018a). Ginsenoside Rh3 inhibits the proliferation of CRC cells in a dose- and time-dependent manner and induces cell apoptosis through upregulation of caspase-3 expression (Cong et al., 2020). Ginsenoside Rb1 decreases the key inflammatory cytokines TNF-α and IL-6 in a CT26 cancer cachexia mouse model (Lu et al., 2020), and ginsenoside Rb2 inhibits the expression of TGF-β1 *in vitro* and *in vivo* (Dai et al., 2019) though downregulation of Smad4 and phosphorylated Smad2/3 expression. Therefore, ginsenosides have the potential to enhance immunotherapy for CRC.

### Research progress in the use of ginsenosides in immunotherapy of esophageal cancer and gastric cancer

The current gold standard treatment for esophageal cancer is adjuvant chemoradiotherapy followed by surgical resection (Vrána et al., 2018), but a low immune response rate and drug resistance are unsolved to PD-L1 blockade. Zhang et al. (2017) observed increased PD-L1 expression in esophageal cancer patients after radiotherapy and in irradiated esophageal squamous cells. High expression of PD-L1 usually suggests that such patients have higher sensitivity to immune checkpoint therapy and several trials are currently addressing the role of immunotherapy in adjuvant radiotherapy and chemotherapy for esophageal/gastroesophageal junction cancer (National Cancer Institute, 2022).

Ginsenoside Rd has been shown to increase the Bax/Bcl-2 ratio, caspase-3 and caspase-9 expression, the downregulation of Cyclin D1, and the inhibition of gastric cancer cell proliferation (Tian et al., 2020). Deng et al. (2020) found that ginsenoside Rh4 has an anti-esophageal cancer effect *in vivo* and *in vitro*. Rh4 significantly inhibits esophageal cancer cell growth by inducing G1 phase arrest. Mechanistically Rh4 may inhibit aerobic glycolysis-related protein expression by targeting Akt, and it also inhibits the expression of PD-L1 through the Akt/mTOR pathway.

## Summary and outlook

There has been significant progress in the treatment of GI tumors through immunomodulation. Targeted drugs, such as immune checkpoint inhibitors and tumor suppressors, are one of the most promising types of therapeutic drugs. However, immune checkpoint inhibitors have only been shown to be efficacious for the treatment of a few types of tumors, such as malignant melanoma and non-small cell lung cancer. The application of these treatments to GI tumors still requires extensive clinical research. In addition, the current popular cancer treatments, including radiotherapy, chemotherapy, targeted inhibitors, and immune checkpoint therapy, still face challenges.

**FIGURE 3:**
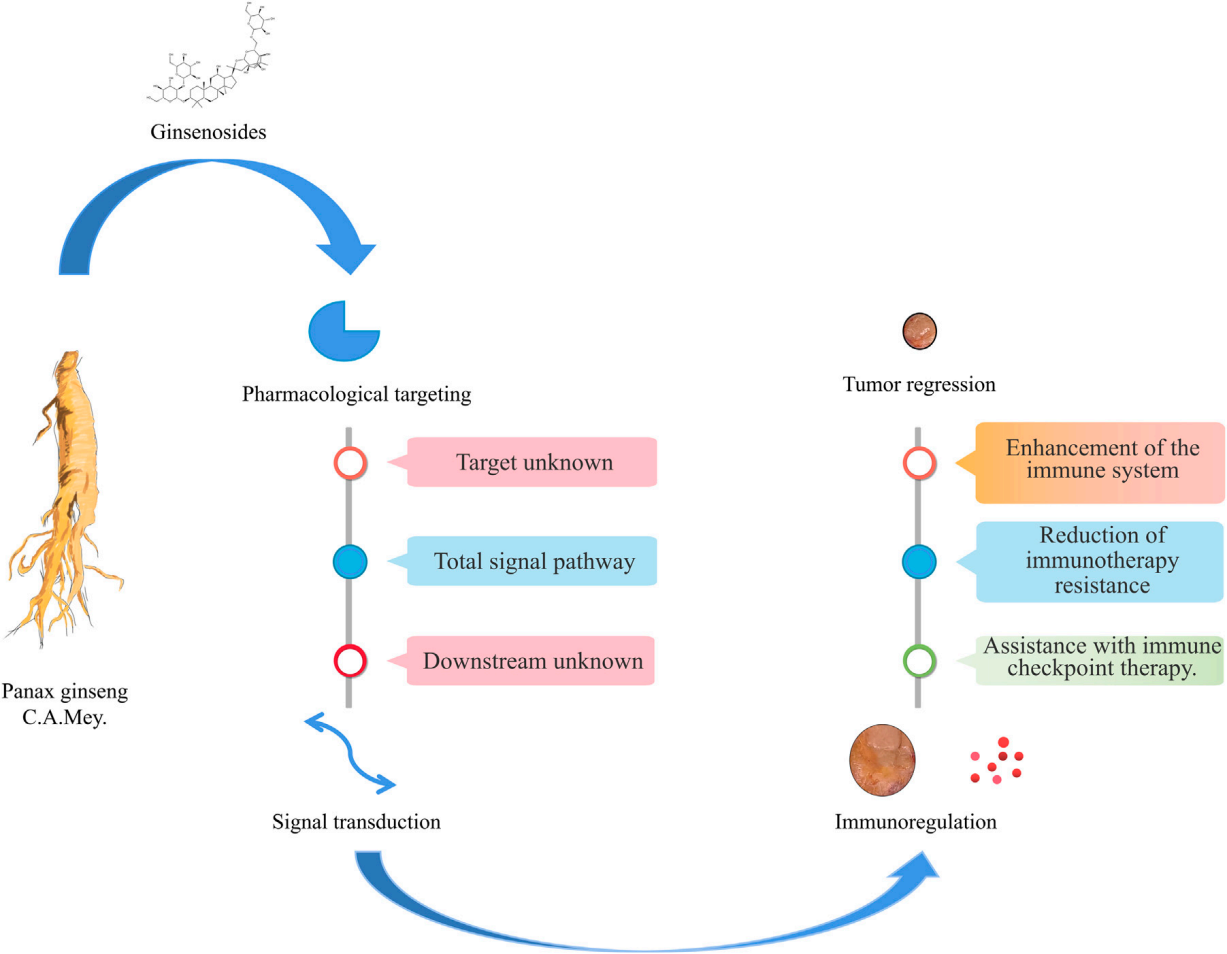
Ginsenosides in immunotherapy of gastrointestinal tumors.

Ginsenosides and related preparations have been used in the clinic for a long time. They have played important roles in the improvement of postoperative fatigue after cancer (Kim et al., 2020b), reversing drug resistance (Jin et al., 2017; Wang et al., 2020a; Yuan et al., 2020), enhancing the efficacy of radiotherapy and chemotherapy (Liu et al., 2017; Liu et al., 2018b; Wang et al., 2020b), and inhibiting tumor cell proliferation and migration (Cai et al., 2013; Kee et al., 2019). Hence, they could potentially complement the first-line anti-GI tumor drugs, improving treatment effects and patient survival times and quality of life (Figure 3 and Table 2). Fuzheng is one of the mainstream anti-tumor strategies of TCM. Its main purpose is to enhance human health without drug toxicity, activate the immune system, and then suppress tumor progression by relying on the immune system (Liang et al., 2009; Zhang et al., 2010). The findings of TCM network pharmacology research in recent years also suggest the great potential of Fuzheng TCM in tumor treatment. The strategy of TCM in treating tumors is similar to immune checkpoint therapy (Zheng et al., 2018). In view of this, we recommend the use of TCM decoctions such as Sijunzi Decoction and Shenling Baizhu powder for the treatment of GI tumors (Zhang and Su, 2008; Xiao and Yang, 2011).

**TABLE 2:** Immune-related pharmacological activities/mechanisms of ginsenosides.

Ginsenoside	Cancer type	Model	Pharmacology	Pharmacological mechanism	References
Rb1	Hypopharyngeal Carcinoma	Xenograft rats	Inhibition of tumor growth with Apatinib treatment	Glycolysis↓; CD3^+^ and CD4^+^ T cells↑ CD4^+^/CD8^+^ T cell ratio↑	Li et al. (2022b)
	Colorectal cancer cachexia	CT-26 cell xenograft BALB/c mice	Recovery of organ weight; reduction of enlarged liver	TNF-α ↓; IL-6↓	Cong et al. (2020)
Rk1	Non-small cell lung cancer	A549 cell	Cancer cell apoptosis	Akt↓, NF-κB↓, PD-L1↓	Hu et al. (2020)
Rb2	Colorectal cancer	HCT116 and SW620 cells	Inhibition of cell growth, adhesion, EMT, and metastasis	TGF-β1↓; Smad4↓; p-Smad2/3↓	Lu et al. (2020)
Rg3	Hepatoma carcinoma	SMMC-7721 and SK-Hep-1 cell	Inhibition of cell proliferation, migration, and invasion.	p-Akt↓, p-PI3K↓, MMP2↓ and MMP9↓, LncRNA HOTAIR↑	Pu et al. (2021)
	Colorectal carcinoma	SW48 and HCT15 cells	Inhibition of cell EMT, migration, and invasion.	IL-6↓, NICD and Hes1↓	Li et al. (2021)
	Non-small cell lung cancer	A549 and A549/DDP cells	Restore the toxicity of T cells to cancer cells	Akt↓, NF-κB↓,PD-L1↓	Jiang et al. (2017)
Rh2	Non-small cell lung cancer	C57BL/6J mice with non-small cell lung cancer	Synergistic antitumor effect with cyclophosphamide; upregulation of the immune deficiency caused by cyclophosphamide	FASN↓, glucocorticoid secretion↓,SREBP-1-FASN interaction ↓	Qian et al. (2019)
Rh4	Esophageal cancer	Eca109 and KYSE150 cells	Growth inhibition by inducing G1 phase arrest	AKT/mTOR↓, PD-L1↓, aerobic glycolysis ↓	Deng et al. (2020)

TCMs are a treasure trove of screened natural drugs, and the exploration of these drugs will contribute to modern anti-tumor strategies. In the overall process of drug development, ginsenosides are still in the early stage. Many ginsenosides have not been optimized or undergone toxicological evaluation, and few clinical trials have been conducted. The research on ginsenoside regulation of immunity, like that on many TCM compounds widely used in clinical practice, has mostly focused on the development of small molecules and preliminary validation of efficacy. Our understanding of the synergistic effect and mechanism of TCM compounds is still relatively limited. Researchers have not found the biomarkers as a standard for the use of ginsenosides in treatment. In an alternative medicine clinical setting, doctors decide whether to use ginseng according to whether the patient has Qi deficiency (cancer-related fatigue) (Hsu et al., 2012; Yang et al., 2016; Kim et al., 2020b). The modernization of ginsenoside preparations is still in its early stage, and a few successes have been achieved. One example of such a success is Ginsenoside Rg3, named the “Shenyi capsule,” which has passed phase IV clinical trials and has been put into clinical use (Huang et al., 2009). Mechanism studies have shown that Rg3 inhibits the growth and angiogenesis of tumor endothelial cells and can significantly downregulate the expression of VEGF and endothelial marker CD31 (Liu et al., 2018b; Li and Qu, 2019). Therefore, the National Comprehensive Cancer Network clinical practice guidelines in oncology (Chinese Version) lists Shenyi capsule as a first-line drug for combined anti angiogenesis treatment and chemotherapy (National Comprehensive Cancer Network, 2006).

Pharmacology research into ginsenosides should be closely combined with frontier technology to interpret TCM from the latest perspective. The advantages of high-throughput technologies such as CyTOF (Sufi et al., 2021) and single-cell sequencing (Tang et al., 2009; Ramsköld et al., 2012; See et al., 2018; Sun et al., 2014; Paik et al., 2020) lie in the high resolution and sequencing depth. These methods can accurately assess cell heterogeneity, and even monitor the whole process of cell development and differentiation and detect protein levels on a single-cell scale. These technologies provide the possibility of obtaining a better understanding of the components in TCMs (Ai et al., 2019; Yang et al., 2022). For example, in a recent study, a non-targeted metabolomics strategy was used to explore the targets of ginsenoside Rg1 in Alzheimer’s disease. Fourteen potential metabolites involved in ten metabolic pathways including linoleic acid metabolism, arachidonic acid metabolism, tryptophan metabolism, and sphingolipid metabolism were affected by Rg1 (Li et al., 2019b). High-throughput virtual screening was used to explore ginsenoside Rb2 binding to TGF-β1 protein in colon cancer (Dai et al., 2019). High-throughput CRISPR screening identified PMAIP1 as resistant and WASH1 as sensitive to ginsenoside CK in a HeLa autophagic cell death model (Yang et al., 2020). These findings have greatly improved our understanding of the pharmacological mechanism of ginsenosides.

At present, research into the synergistic effects of ginsenosides and immunotherapy is still in its infancy. Our understanding of these synergistic effects and the mechanism of action of ginsenoside as a tumor suppressor is still relatively limited. There is an urgent need for in-depth exploration of targets such as CTLA-4 and PD-1/PD-L1 and related signaling pathways such as Akt (Deng et al., 2020; Yuan et al., 2020) and EGFR (Li et al., 2018). New clinical trials on immunotherapies targeting LAG3, TIM3, TIGIT, and BTLA are underway (Andrews et al., 2017; Zhang et al., 2018; Kraehenbuehl et al., 2022). Hopefully, with the development of clinical trials and combination medication research, ginsenosides may become a powerful ally in immunotherapy.
